# The Evolving Field of Risk Communication

**DOI:** 10.1111/risa.13615

**Published:** 2020-10-20

**Authors:** Dominic Balog‐Way, Katherine McComas, John Besley

**Affiliations:** ^1^ Department of Communication Cornell University Ithaca NY USA; ^2^ Department of Advertising and Public Relations Michigan State University East Lansing MI USA

**Keywords:** Interdisciplinary, literature review, risk communication, SRA anniversary

## Abstract

The 40th Anniversary of the Society for Risk Analysis presents an apt time to step back and review the field of risk communication. In this review, we first evaluate recent debates over the field's current state and future directions. Our takeaway is that efforts to settle on a single, generic version of what constitutes risk communication will be less productive than an open‐minded exploration of the multiple forms that comprise today's vibrant interdisciplinary field. We then review a selection of prominent cognitive, cultural, and social risk communication scholarship appearing in the published literature since 2010. Studies on trust in risk communication *messengers* continued to figure prominently, while new research directions emerged on the opportunities and critical challenges of enhancing transparency and using social media. Research on *message attributes* explored how conceptual insights particularly relating to framing, affective and emotional responses, and uncertainty might be operationalized to improve message effectiveness. Studies consistently demonstrated the importance of evaluation and how varying single attributes alone is unlikely to achieve desired results. Research on risk communication *audiences* advanced on risk perception and multiway engagement with notable interest in personal factors such as gender, race, age, and political orientation. We conclude by arguing that the field's interdisciplinary tradition should be further nurtured to drive the next evolutionary phase of risk communication research.

## 1 INTRODUCTION

**Fig 1:**
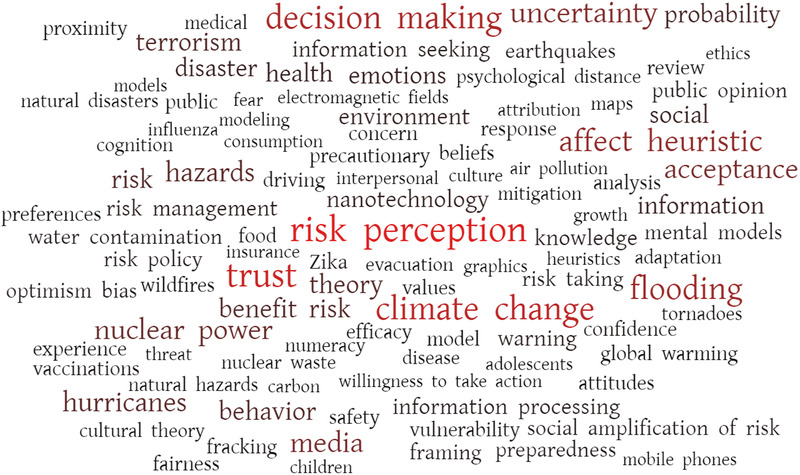
Word cloud displaying article keywords (*N* = 1,370) collected from every risk communication paper published in *Risk Analysis* between January 2010 and December 2019 (*N* = 329). Larger and redder keywords appeared more frequently. Keywords appearing fewer than three times were excluded. Those that mean the same thing, such as flooding and floods, were given a common label (e.g., flooding). Generated using WordItOut.com.

Since its inception in August 1980, the Society for Risk Analysis (SRA) has played an influential role in the evolution of risk communication research (Aven, 2018; Thompson, Deisler, & Schwing, 2005). SRA's flagship journal, *Risk Analysis*, has published a raft of risk communication scholarship (Greenberg et al., 2012, 2020; Thompson et al., 2005), including oft‐cited seminal articles on the social amplification of risk framework (Kasperson et al., 1988), trust (Siegrist & Cvetkovich, 2000; Slovic, 1993), the development of risk communication (Fischhoff, 1995), and the importance of affect in risk perception (Slovic, Finucane, Peters, & MacGregor, 2004), to name a few. Between January 2010 and December 2019 an estimated 329 risk communication articles were published in the journal.^1^ By collecting 1,370 keywords, Fig. 1 provides a simple way of depicting the diversity of these contributions. While risks like climate change, “natural” hazards/disasters (esp. flooding, hurricanes, earthquakes), nuclear power, and nanotechnology received substantial interest, research on risk perception, trust, affect, acceptability, uncertainty, media, and decision making figured prominently (Fig. 1).

The risk communication literature is by no means confined to articles published in *Risk Analysis*. Rather, our knowledge is dispersed across an interdisciplinary and fragmented field of research and practice (Bostrom, Böhm, & O'Connor, 2018; Löfstedt & 6, 2008; Rickard, 2019; Wardman, 2008). Risk communication scholars with contrasting interests, knowledge, and research traditions publish in diverse academic journals. Authors’ disciplinary backgrounds range from communication, sociology, geography, and anthropology, to psychology, and the decision sciences (Fischhoff & Broomell, 2020; Rickard, 2019), among many others. Our knowledge extends to the wide‐ranging gray literature and unpublished practical experience (OECD, 2016; World Bank, 2013); yet the linkage between scholarship and practice cannot be taken for granted, as empirical evidence shows that many practitioners remain disconnected from academic knowledge (Boholm, 2019).^2^

In seeking to comment on, and evaluate, the latest developments in risk communication research, this literature review is organized around two themes that dominated the published literature since 2010. We first review recent debates over the current state and future directions of the field. These debates include various perspectives on how the field has been shaped and reshaped over time, and we concentrate on evolving conceptualizations of “risk” in society and how risk communication practice could and should be constituted. Next, we review a selection of prominent cognitive, cultural, and social risk communication scholarship, focusing on risks to human health and the environment. Considering the wealth of research published since 2010, we are unable to include every noteworthy study. We also chose to focus on the last 10 years to provide more space for commenting on and evaluating the most recent developments and because other excellent reviews already exist for earlier developments in the field. This includes reviews on the first 30 years since SRA's establishment; concepts like trust, culture, and uncertainty; methods such as mental models and risk perception measures; and the underlying rationales and theoretical orientation of the field.^3^

## 2 CONCEPTUALIZING RISK COMMUNICATION RESEARCH

The boundaries of risk communication research and practice are not static but have been shaped and reshaped over time, partly through our evolving conceptual understating of “risk” in society (Boholm & Corvellec, 2011, 2014; Douglas, 1985, 1992; Hilgartner, 1992; Leiss, 1996; McComas, 2006; Rosa, 1998). During the early 1980s, many risk communication researchers and practitioners treated risk as the outcome of “objective,” “expert” risk assessment processes, whereas “lay” perspectives were considered subjective and irrational (Boholm, 2009; Fischhoff, 1995; Leiss, 1996; National Research Council, 1989). The field was largely defined by attempts to align “lay” perspectives with those of “the experts” with the expectation that this would change lay behavior (Frewer, 2004; Hagendijk & Irwin, 2006; Hilgartner, 1990), or what came to be known as the deficit model (Gregory & Lock, 2008). Scholars’ recognition that risk judgments are not limited to risk assessments, and that risk encompass both objective and subjective qualities, contributed to fundamentally reshaping the field and its boundaries (Cvetkovich & Löfstedt, 2013; Leiss, 1996; McComas, 2006). Risk began to be widely recognized as a social construct (Leiss, 1996), whereby “risk judgments are, to some degree, a by‐product of social, cultural, and psychological influences” (McComas, 2006, p. 76; see Slovic, 1999, for a discussion). As our understanding of “risk” in society continues to evolve, the field of risk communication continues to evolve as well (Boholm, 2009; Boholm & Corvellec, 2011, 2014).

Our review suggested that debates about our understanding of risk in society were not the only elements shaping risk communication research. Rather, an evolution in thinking about how risk communication practice could and should be constituted, as well as developments in related fields, also was reflected in the scholarship. In particular, the risk communication community at large have gradually replaced the deficit model with “multi‐way”^4^ approaches to risk communication (Slovic, 1999; Webler & Tuler, 2018), emphasizing the importance of engaging audiences through meaningful dialog and deliberation (see Pidgeon, 2020, for a discussion), although Renn (2014, p. 1277) notes that the deficit model “maintains a substantial following in industry and in state, local, and federal government.” Crucially, then, the last decade saw an upsurge in debate over “What constitutes *effective* risk communication?” (Árvai and Rivers III, 2014; Rickard, 2019), including a thought‐provoking article by Roger Kasperson (2014) that proceeded eight commentaries (Árvai, 2014; Bostrom, 2014; Fischhoff, 2014; McComas, 2014; Pidgeon, 2014; Renn, 2014; Siegrist, 2014; Wardman, 2014). Noting that effectiveness refers to the degree to which a *desired result* is achieved, the debate hinged on various normative, instrumental, and substantive arguments for engaging in multiway risk communication (Demeritt & Norbert, 2014; Dietz, 2013; Fiorino, 1990; McComas, Árvai, & Besley, 2009; Pidgeon, 1998, 2020; Wardman, 2008).

*Normative* arguments stressed no particular desired end result for effective multiway risk communication (Wardman, 2008). Practice is understood as having an intrinsic value as the right thing to do in and of itself in a democratic society (Dietz, 2013; Pidgeon, 2020). In exploring the perspectives of marginalized Aboriginal and/or northern peoples in Canada, Jardine and Driedger (2014) note that the democratic ideal of empowerment can only be achieved when individuals have a level of knowledge and awareness that enables meaningfully engagement. Others argued that actors such as governments, businesses, and scientists have an obligation to inform honestly, precisely, with audience relevance, and while specifying uncertainties about conclusions (see Keohane, Lane, & Oppenheimer, 2014). Individuals are often considered the best judges of their own interests from a normative perspective, or as Siegrist (2014, p. 1241) commented: “I do not know what decisions other people should make in order to successfully strive to be happy. I hope, however, that good risk communication will help consumers to make better decisions.”

*Instrumental* arguments centered on effective multiway risk communication as a resource or means for individuals, groups, or organizations to help achieve desired results (Wardman, 2008). In evidence‐based guidance materials for the U.S. Food and Drug Administration (Fischhoff, Brewer, & Downs, 2011), Brewer (2011) outlined the strengths and weaknesses of three broad risk communication goals: sharing information, changing beliefs, and changing behavior (also see Bostrom et al., 2018). An instrumental behavior change goal, for instance, requires that risk communicators know the best course of action (Brewer, 2011). Pharmaceutical risk regulators might create a *Safety Communication* to stop patients consuming a medicine now deemed unsafe (Way, Blazsin, Löfstedt, & Bouder, 2017). Hurricane risk communicators often have a public duty or an ethical obligation to protect residents by communicating evacuation orders. During the COVID‐19 pandemic, most governments sought to persuade the public to physically distance as much as possible to reduce virus transmission and fatalities (Balog‐Way & McComas, 2020). Other desired goals for risk communicators include fostering trusting behavior or enhancing legitimacy such as through multiway dialog, consensus building, and conflict resolution (Jardine & Driedger, 2014; Pidgeon, 2020), although there also may be value in distinguishing the more immediate *objectives* of communication in terms of desired evaluative beliefs, feelings, and frames from the longer term *outcomes* on goal behaviors (Bennett, Dudo, Yuan, & Besley, 2019). Notably, most research made clear that not all instrumental goals are desirable in all circumstances and the end result does not necessarily justify the means (e.g., lying to an audience to get them to accept a technology, hazard, or activity).

*Substantive* arguments stressed the ability of effective multiway risk communication to generate new insights and improvements in the quality of available knowledge by “opening up the framing and evaluation of decisions and options” (Pidgeon, 2020; Wardman, 2008). Public participation and deliberation, for example, continued to be promoted as a way of substantively engaging “outsiders” to reflect useful noninstitutionalized knowledge and experience back to the risk characterization and management process (Collins & Evans, 2002; Demeritt, 2015; Leiss, 2014; Pidgeon, Harthorn, Satterfield, & Demski, 2017; Renn, 2010; Tuler, Dow, & Webler, 2017). As Webler and Tuler (2018, p. 5) put it: “participants bring different types of knowledge and experience to the table” including “valid epistemological contributions.” Others explored how social media can provide new opportunities for substantively engaging in multiway dialog through dynamic, interactive features like following, commenting, liking, and retweeting, which enable the creation and exchange of “content” (Neeley, 2014; Sutton & Veil, 2017). In the immediate aftermath of various disasters, for example, many shared “where they are, what they see, how they feel, and if they need help” (Neeley, 2014, p. 150). Disaster response risk communicators can engage with such “audiences” to help survivors by gathering information and connecting individuals, all the while improving the quality of knowledge about the threat itself (Neeley, 2014; Sutton & Veil, 2017).

Recent perspectives on how risk communication could or should be constituted, including normative, instrumental, and substantive arguments, reflect to some extent the historical context that gave rise to the field. Historical processes such as path dependency, sequencing, timing, critical junctures, and unintended consequences (see Pierson, 2004) have all fundamentally shaped how scholars differentially understand the field today. Many contributors (e.g., Kasperson, 2014) referred to the 1986 First Conference on Risk Communication, the National Research Council's (1989) publication on *Improving Risk Communication*, and efforts in the 1980s more generally to apply emerging risk perception findings to risk communication practice. In contrast, histories of risk communication in the pharmaceutical area rarely cited these critical junctures that came to define the environmental/technological areas. Way et al. (2017), for instance, traced contemporary pharmaceutical benefit–risk communication to the 1997 Erice Declaration on *Communicating Drug Safety Information*, and the rise of concepts like shared patient–doctor decision making, informed consent, informed choice, and the right to challenge the authority of physicians. Research examining the citing of a nuclear waste repository at Yucca mountain (e.g., Flynn, Slovic, & Mertz, 1993) strongly influenced the development of multiway environmental/technological risk communication but is rarely cited in pharmaceutical, crime, or financial risk communication histories. Similarly, risk communication scholars have brought ideas like public participation from other areas of scholarship (e.g., Arnstein, 1969; Mansbridge, 1983; Pateman, 1970). The field of risk communication, including its constitution and boundaries, thus can be understood as systematically historically situated in a temporal sequence of events and processes (see Pierson, 2004; Plough & Krimsky, 1987).

In sum, our review of these recent perspectives lead us to conclude that there will never be a single, generic version of how risk communication could and should be constituted, or a single historical narrative (Demeritt & Norbert, 2014; Rickard, 2019; Wardman, 2008, 2014). Various valid reasons for engaging in practice mutually coexist, although not all reasons are acceptable, ethical, or effective in every circumstance. An alternative endeavor that might constructively advance the field would be both to explore and debate the strengths and weaknesses of different forms of risk communication in light of their intended outcomes (Demeritt & Norbert, 2014; Rickard, 2019; Wardman, 2008, 2014). Wardman (2008), for example, posited four idealized forms undergirded by the aforementioned normative, instrumental, and substantive rationales. In the context of flooding risk communication, Demeritt and Norbert (2014) found that when different forms are not acknowledged or understood, then efforts to improve practice will most likely produce inconsistent and contradictory approaches. Rickard (2019) distinguished between pragmatic and constitutive^5^ functions of risk communication, arguing that research on both are important for addressing 21^st^‐century societal risks. Crucially, future debate is needed on the relative strengths and weaknesses of different coexisting forms of risk communication including their underlying rationales. In turn, greater conceptual clarity about the continually evolving field, its boundaries, and future directions can be realized.

## 3 INFLUENTIAL RESEARCH DIRECTIONS

This next section spotlights three prominent research areas that undergird the pragmatic function of risk communication as a key component of formal risk analysis: messengers, message attributes, and audiences (see Rickard, 2019). We recognize that these are imperfect groupings, and the pragmatic risk communication literature can and has been subdivided in other ways.^6^ For this review, *messengers* refer to the individuals, groups, and organizations purposefully engaging in risk communication;^7^ *message attributes* refer to the qualities and inherent characteristics of verbal, written, or recorded risk communication; and *audiences* refer to various intended message recipients and incorporates engagement through multiway dialog (Pidgeon, 2020).

### 3.1 Messengers

The last decade was bookended by two noteworthy reviews on trust^8^ in risk communication messengers (Earle, 2010; Siegrist, 2019), revealing a now large but complicated literature. Common research themes revealed by these reviews were trust models, types, and dimensions; the importance of trust in varying contexts; the stability of trust; and the relationship between trust and its consequences such as for risk perception, benefit perception, and behavior (Earle, 2010; Siegrist, 2019). A complementary research strand concentrated on procedural and interpersonal fairness perceptions (Besley & McComas, 2014; Dixon, McComas, Besley, & Steinhardt, 2016; Visschers & Siegrist, 2012; Webler, 2014). Research showed that trust and fairness played a central role for messengers in virtually every context studied, ranging from climate change (Smith & Mayer, 2018), nuclear power (Besley & Oh, 2014; Visschers & Siegrist, 2013), and autonomous vehicles (Liu, Yang, & Xu, 2019), to pharmaceutical (Balog‐Way, Evensen, Löfstedt, & Bouder, 2019), food (McComas, Besley, & Steinhardt, 2014), and chemical (Saleh, Bearth, & Siegrist, 2019) risk communication. Trust is highly valued by messengers as audiences must rely on *credible* sources to help inform risk judgments, preferences, and choices, especially when knowledge of a technology, hazard, or activity is low (Siegrist, 2019; Tuler & Kasperson, 2014). During recent infectious disease outbreaks, like swine flu, the 2014 Ebola outbreak, and the COVID‐19 pandemic, studies found correlations between trust in messengers and the public's (1) perceived disease severity, (2) perceived virus transmissibility, (3) information seeking behavior, and (4) willingness to adopt interventions such as recommended hygiene practices and physical distancing measures (Blair, Morse, & Tsai, 2017; Cairns, Andrade, & MacDonald, 2013; Fancourt, Steptoe, & Wright, 2020; Fischhoff, Wong, Garfin, Holman, & Cohen Silver, 2018; Ploh & Musil, 2020; Vinck, Pham, Bindu, Bedford, & Nilles, 2019).

While building and maintaining trust in messengers remains a key focus of inquiry, the literature suggests no simple one‐size‐fits‐all solutions to reverse the long‐term declining levels of trust observed in many countries and contexts (Tuler & Kasperson, 2014). Empirical research recommends varying approaches when trust is high or low and during different stages of an acute event like a product recall or disease outbreak (Rickard, McComas, Clarke, Stedman, & Decker, 2013). Therefore, regularly testing baseline trust levels is key (Balog‐Way et al., 2019; Löfstedt & Bouder, 2017). Structural and procedural approaches include developing long‐term trusted relationships with key actors like journalists, local leaders, and other influential messengers; designing clearly structured risk communication systems to help coordinate activities and interactions with public audiences; building baseline and surge capacities like hiring permanent and trained messengers, while involving them at the strategic level of decision making; and engaging audiences in meaningful multiway dialog (Balog‐Way & McComas, 2020; Bouder, Way, Löfstedt, & Evensen, 2015; Löfstedt, 2013a, 2019; National Academy of Sciences, 2016a; Rickard et al., 2013; Tuler & Kasperson, 2014). Rickard et al. (2013) explored risk communication responses to a plague death event in the Grand Canyon, USA, and identified various precrisis, crisis, and postcrisis actions. “Pre‐accumulated” interagency trust, for example, can help build public trust by enabling messengers to speak with one voice, coordinate activities together, and share information quickly (Rickard et al., 2013). Also, just as the trust literature highlights the value of trust as the outcome of semidistinct perceptions of factors such as decisionmakers’ benevolence (i.e., warmth), integrity, and ability (i.e., competence) (Fiske & Dupree, 2014; Hendriks, Kienhues, & Bromme, 2015), the literature on procedural justice as fairness highlights the value of decision‐making processes that give audiences a voice and treats them with respect, while ensuring access to available information (Besley & McComas, 2014).

Many messengers now view transparency as essential for (re)building trust^9^ (Carpenter, 2017; Dixon et al., 2016; Dudley and Wegrich, 2016; Löfstedt & Bouder, 2014; O'Connor, 2016; Way, Bouder, Löfstedt, & Evensen, 2016). Enhancing transparency is expected to result in better informed audiences, who will be well‐placed to judge the trustworthiness of messengers positively, while opaque messengers will be perceived as misleading, misinforming, or concealing information (Löfstedt & Bouder, 2014; Way, 2017, pp. 60–64). Similarly, perceived “openness” is understood as an important messenger characteristic for ensuring risk communication processes are perceived fairly (Besley & McComas, 2014). However, the emerging literature reveals that various transparency policies exist, which can produce serious unwanted side effects and varying positive, limited, and negative effects on trust (Bouder et al., 2015; Cucciniello, Porumbescu, & Grimmelikhuijsen, 2017; Meijer, Hart, & Worthy, 2018; Way, 2017). For example, by surveying the perspectives of European patients (*N* = 1,010) and doctors (*N* = 1,005), two studies (Löfstedt, Way, Bouder, & Evensen, 2016; Way et al., 2016) found that few patients had even heard of the pharmaceutical regulators seeking to build trust, the large majority of doctors believed it is a bad idea to publicly release unverified safety information, and both groups self‐reported low knowledge of how drugs are approved. As a prerequisite for building trust, *effective* transparency requires that audiences can receive, process, digest, and use information made available (Heald, 2006; Keohane et al., 2014; Way, 2017). Many messengers, however, became captivated by quantity over quality over the last decade, resulting in the publication of enormous volumes of data online, which can result in even the most expert audiences becoming less informed and more confused (Löfstedt et al., 2016; O'Neill, 2006). Moreover, if messengers reconceptualize transparency as a risk communication process, then decades of research can be usefully applied to understanding the relative strengths and weaknesses of various forms, to help identify the most promising, and least damaging, approaches for (re)building trust (Way, 2017).

The marked increase in the popularity and diversity of social media channels over the last 10 years provided messengers with new opportunities and expanded the range of people who can send messages to narrow and broad audiences. Messengers can now deliver messages faster to both broader and more targeted audiences, while capitalizing on various forms of content like audio‐visuals, pictures, and text (Rains, Brunner, & Oman, 2015; Schultz, Utz, & Göritz, 2011). During a heavy snowstorm in 2010 and rioting in 2011, Panagiotopoulos, Barnett, Bigdeli, and Sams (2016, p. 86) found that U.K. local authorities posted over 10,000 messages on Twitter that sought to “provide official updates, encourage protective behaviour, increase awareness, and guide public attention to mitigating behaviours.” Messengers can open dialog with audiences by using interactive social media features, such as replying to comments or using real‐time videos, which have been suggested as a new way of engaging in participatory multiway risk communication and building trusted relationships with influential opinion leaders and groups (Rains et al., 2015). Although studies are limited, researchers recognize that the affordances provided by different forms of social media like blogs, microblogs, social network websites, discussion forums, and video and photo‐sharing websites allow for different types of effects (Binder, 2012; Panagiotopoulos, Barnett, Bigdeli, & Sams, 2016; Rains et al., 2015; Regan, Raats, Shan, Wall, & McConnon, 2016). Messengers also can use social media to facilitate substantive improvements in risk‐related knowledge (Rains et al., 2015). Social media has been successfully used by messengers, for example, to crowdsource knowledge about missing persons, obtain vital “on the ground” response and recovery information during crises, and support collective forms of coping (Demuth et al., 2018; Lambert, 2020). Furthermore, social media has helped shape a new digital information environment where “backchannel” peer‐to‐peer communication has become more visible, influential, and interactive, thus challenging the dominance of traditional risk communication messengers such as risk managers, journalists, and other professional information brokers (see Sutton & Veil, 2017, for a discussion; Sutton et al., 2014).

Social media, however, comes with challenges beyond attempts to capitalize on new opportunities and avoid unintended consequences. Messengers may have to compete for attention as valued sources of information, while their audiences are awash with near instantaneously distributed information (Mayorga et al., 2020; Overbey, Jaykus, & Chapman, 2017). Loosely referred to as “fake news”, messengers have to keep pace with increasing volumes of *misinformation*, false information shared without intent to harm; *mal‐information*, accurate information taken out of context with the intent to harm; and *dis‐information*, knowingly false information shared with the intent to harm (Del Vicario et al., 2016; Mayorga et al., 2020; Wardle & Dearkhshan, 2017). By investigating ∼136,000 stories distributed on Twitter from 2006 to 2017, Vosoughi, Roy, and Aral (2018) found that false versus true information spread significantly farther, faster, deeper, and more broadly, especially when information was perceived as “novel” and evoked fear, disgust, and/or surprise. To examine social media effects, several researchers applied the social‐mediated crisis communication model (Jin, Liu, & Austin, 2014), or social amplification of risk framework (Binder, 2012; Comrie, Burns, Coulson, Quigley, & Quigley, 2019; Fellenor et al. 2018; Panagiotopulos et al. 2016; Strekalova & Krieger, 2017; Wirz et al., 2018; Zhang, Xu, & Zhang, 2017). The emerging literature highlights that audiences are not passive information recipients (Neeley, 2014) and there is a crucial distinction between audiences’ exposure to social media content—including false, inaccurate, or misleading information—and influences on attitudes and behavior. Although social media research is growing rapidly such as with large‐scale studies exploring misinformation using approaches such as inoculation theory (Basol, Roozenbeek, & Linden, 2020; Roozenbeck & van der Linden, 2019; van der Linden, Panagopoulos, & Roozenbeek, 2020), much remains unknown about the role and consequences of social media in risk communication, and additional theory‐building and adaptation efforts are needed (Lazer et al., 2018; Rains et al., 2015).

Another area that evolved over the last decade centered on how to encourage messengers like scientists (Bennett et al., 2019) or regulatory authorities (Fischhoff, 2017) to communicate more proactively and effectively. Some early research focused largely on increasing the quantity of risk communication activity (Besley & Oh, 2014; Poliakoff & Webb, 2007). More recent efforts turned to improving the quality of risk communication, often through in‐person training (Besley, Dudo, Yuan, & AbiGhannam, 2016; Miller, Fahy, & Team, 2009; Rodgers et al., 2018) and developing evidence‐based guidance materials (e.g., Baron, 2010; Fischhoff et al., 2011). One aspect of improving the quality of risk communication revolved around helping trainers and messengers move beyond simply discussing communication tactics (e.g., speaking clearly, telling stories, considering nonverbal cues), to thinking more about effectiveness, including carefully selecting objectives beyond conveying scientific knowledge (e.g., fostering trustworthiness or beliefs) and clarifying overall goals (e.g., behavior change, enhancing legitimacy, or learning from stakeholders) (Besley, Newman, Dudo, & Tiffany, 2020; Brewer, 2011; Dudo & Besley, 2016), as well as structural and procedural organizational changes like making risk communication evaluations a standard operating procedure (Fischhoff, 2019).

### 3.2 Message Attributes

Framing of various kinds plays a central role in all messages, regardless of whether frames were purposefully or intuitively considered (Nisbet, 2015). Nisbet (2015, p. 216) defines frames in a sociological sense as “interpretative story lines that set a specific train of thought in motion, communicating why an issue might be a problem or pose a threat or what might be responsible for it, and what should be done about it.” Frames compared using this approach over the last decade include humans versus the environment, gains versus losses, closeness versus distance, threats versus benefits, and economics versus health, to name a few (McComas, Schuldt, Burge, & Roh, 2015; Myers, Nisbet, Maibach, & Leiserowitz, 2012; Schuldt, McComas, & Burge, 2017a; Schuldt, Rickard, & Yang, 2018; van Boven, Ehret, & Sherman, 2018). In these cases, different types of content are used to suggest thinking about underlying issues in different ways. In contrast, adopting a psychological focus, some have explored how variations in equivalence data or terms such as fracking for shale oil (Clarke et al., 2015; Evensen, Jacquet, Clarke, & Stedman, 2014), genetic engineering for genetic modification (Zahry & Besley, 2019), or global warming for climate change (Schuldt, Enns, & Cavaliere, 2017b)—can also have consequences for risk perception, benefit perception, and/or behavioral intentions. In exploring the concept of “intersecting frames,” Schuldt et al. (2017a) found that framing climate change as both (1) a public health issue and (2) “climate change” (rather than “global warming”) increased skepticism among political conservatives, even though past research on these two frames in isolation showed decreased skepticism. More generally, framing choices can fundamentally alter the nature of a risk issue by setting the context for risk perceptions, conversations, and disputes (Boholm, 2009; Nisbet, 2015). During the construction of the Hallandsås railway tunnel in Sweden, Boholm (2009) found that the Swedish Rail Administration and local stakeholders framed the risk dispute wholly differently including what was considered the object at risk and risk object.

Studies also explored how frames are likely to be ignored when they fail to draw relevant connections or lack personal significance (Nisbet, 2015). Research has particularly expanded on whether using framing techniques to shape the psychological distance of climate change, including spatial, temporal, social, and/or hypothetical dimensions, can lead to greater engagement by making the issue more local, immediate, relevant, and real (Maibach, Nisbet, Baldwin, Akerlof, & Diao, 2010; McDonald, Chai, & Newell, 2015; Schuldt et al., 2018; Spence, Poortinga, & Pidgeon, 2012). Some survey and experimental studies suggest that making climate change feel *closer* may increase willingness to restrict energy use (Spence et al., 2012), heighten actions for government support (Milfont, Evans, Sibley, Ries, & Cunningham, 2014), change‐specific climate‐mitigation behaviors (Broomell, Budescu, and Por, 2015), and increase levels of concern (Jones, Hine, & Marks, 2017). However, other studies produced mixed results (e.g., Manning et al., 2018), with authors expressing methodological concerns over the use of surveys to assess causality (Schuldt et al., 2018) and that reducing psychological distance is unlikely to increase support or policy engagement on its own (Brügger, Morton, & Dessai, 2016; Schuldt et al., 2018). Some risk communication framing research has been critiqued more generally for lacking theoretical clarity across different studies (Cacciatore, Scheufele, & Iyengar, 2016; D'Angelo et al., 2019; Krippendorff, 2017), oversimplifying “real world” communication where audiences are exposed to multiple intersecting frames (Schuldt et al., 2017a), and struggling to keep up with rapid changes in media systems (Nisbet, 2015).

A sizable body of message attributes research sought to capitalize on advances in “risk‐as‐feelings” research (Kahneman, 2011; Loewenstein, Weber, Hsee, & Welch, 2001; Slovic et al., 2004), which strongly suggests that risk communicators should attend to the affective and emotional components of messages (also *see* risk perception and affect in Section 3.3) (Roeser, 2012; Tannenbaum et al., 2015). As Slovic (2010) argues, “…people look to their positive and negative feelings to guide their evaluation of an activity's risks and benefits” and “feelings serve as an important cue for benefit/risk judgements and decisions.” Some studies explored the role of narratives and stories in appealing to discrete emotions, as well their ability to make messages easier to comprehend and more engaging (Dahlstrom, 2014; Margolis, Brewer, Shah, Calo, & Gilkey, 2019; Niederdeppe, Shapiro, & Porticella, 2011; Niederdeppe, Shapiro, Kim, Bartolo, & Porticella, 2014). Janssen, van Osch, de Vries, and Lechner (2013), for instance, found that narrative versus nonnarrative messages resulted in female sun bed users reporting higher feelings of skin cancer risk. Numerous other studies examined the role of feelings for various visualizations like photographs, pictograms, graphs, maps, videos, and augmented/virtual reality (Kundu & Nawaz, 2017; Niederdeppe, Roh, & Dreisbach, 2016; Rickard, Schuldt, Eosco, Scherer, & Daziano, 2017a; Xie, Wang, Zhang, Li, & Yu, 2011). In a literature review, Downs (2014) concluded that videos can be powerful risk communication tools for changing behavior, although they can be costly and may produce unintended consequences. Furthermore, a handful of studies explored the role of message interactivity such as the use of “serious games,” where the primary purpose is not pure entertainment (Roozenbeek & van der Linden, 2019; Solinska‐Nowak et al., 2018). In the disaster risk communication area, Solinska‐Nowak et al. (2018) found that serious games may assist efforts to trigger empathy, raise awareness of “natural” hazards, and persuade audiences to undertake preventative measures.

Feeling positive “discrete emotions” like happiness and amusement also received growing attention. Although many risk communication researchers historically recommended against the use of humor (e.g. due to concerns about trivializing risk issues; Ferrante, 2010), the topic received noteworthy attention over the last decade, especially in health, environmental, and precrisis risk domains (Blanc and Brigaud, 2014; Boykoff & Osnes, 2019; Moyer‐Gusé, Mahood, & Brookes, 2011; Skurka, Niederdeppe, Romero‐Canyas, & Acup, 2018). Some found that humor can, indeed, have negative effects such as by distracting audiences from the central risk message (Hansmann, Loukopoulos, & Scholz, 2009), or damaging intentions to uptake recommended behavior (Skurka et al., 2018). Humor, however, can be highly beneficial when executed effectively in the right contexts. In reviewing the literature on humor and climate change, Kaltenbacher and Drews (2020) found that humor can raise awareness such as through cartoons, and memes (Ross & Rivers, 2019); help audiences psychologically cope and deal with negative emotions (Murthy & Gross, 2017); foster greater involvement (Anderson & Becker, 2018); influence perceptions and beliefs (Brewer & McKnight, 2017); help audiences overcome awkward or taboo risk issues (Browne, 2016); function as a learning vehicle (Boykoff & Osnes, 2019); and influence positive behavior changes (Skurka et al., 2018). While research seeking to capitalize on the risk‐as‐feelings literature continues to show promise, empirically tested risk communication applications remain in the nascent stages of development.

The advantages and pitfalls of communicating uncertain risk information, or simply “uncertainty communication,” continued to receive substantial attention (Beck et al., 2016; Fischhoff & Davis, 2014; Löfstedt & Bouder, 2017; Löfstedt, McLoughlin, & Osman, 2017; National Academy of Sciences, 2016b; Osman, Ayton, Bouder, Pidgeon, & Löfstedt, 2019; Osman, Heath, & Löfstedt, 2018; Sahlin & Troffaes, 2017; van der Bles et al., 2019; van Der Bles, van der Linden, Freeman, & Spiegelhalter, 2020). Among other benefits, some argued that uncertainty communication can help build public trust; enhance transparency; enable meaningful multiway risk communication; and increase the legitimacy and credibility of the decision‐making process (see Löfstedt & Bouder, 2017, for a discussion; Beck et al., 2016; Joslyn & LeClerc, 2013; van der Bles et al., 2019). In an experimental study examining the communication of facts, formats, and magnitudes, van Der Bles et al. (2020) found that epistemic uncertainty can be communicated effectively with limited negative effects on trust. Others, however, argue that uncertainty communication may “do more harm than good” (Osman et al., 2018) by inadvertently eroding public trust; reducing confidence in messengers; undermining the decision‐making process or central message; providing new opportunities for misuse or abuse of data/information; and, ultimately, decreasing transparency such as by snowing “outsiders” under confusing and complex risk information (Bostrom et al., 2018; Löfstedt & Bouder, 2017; Way, 2017). The European Food Safety Authority's (EFSA) approach to uncertainty analysis transparency, for instance, has been criticized for poorly communicating uncertain risk information (Löfstedt & Bouder, 2017), with empirical research suggesting public trust will decrease as a result (Löfstedt et al., 2017; Osman et al., 2018), including studies sponsored by the authority (e.g., EFSA, 2019). Notably, most criticisms are not directed at the concepts of transparency or uncertainty communication *per se*. Rather, they stem from concerns over the current methods and approaches adopted by certain practitioners (Osman et al., 2018).

Although the literature on uncertainty communication is mixed, at least three overarching findings can be highlighted. First, different forms of uncertainty, such as deficient, technical, consensus, and scientific, need to be recognized in research and practice (Bostrom et al., 2018; Gustafson & Rice, 2020; National Academy of Sciences, 2016b; van Der Bles et al., 2020). Second, there are no clear‐cut or simple solutions for communicating uncertain risk information effectively, or, as Bostrom et al. (2018) put it: “Deciding how best to communicate uncertain risks quantitatively is not simple, as there are few generally accepted rules of thumb.” Third, and perhaps most importantly, positive and/or negative effects related to trust, legitimacy, and transparency, are strongly influenced by how well uncertain risk information is communicated in the first place (Balog‐Way & McComas, 2020; Fischhoff & Davis, 2014; Jensen et al., 2017; Löfstedt & Bouder, 2017). Evidence‐based uncertainty communication, that mitigates negative effects and maximizes potential benefits, demands evaluation and commitment from practitioners (Fischhoff & Davis, 2014; Osman et al., 2018; Rakow, Wright, Spiegelhalter, & Bull, 2015; Schwartz & Woloshin, 2013). To advance this area, some have argued for a systematic literature review undergirded by a sophisticated organizing framework, which recognizes different forms of uncertainty communication and can guide future studies by identifying what type of uncertainty could or should be communicated in practice, how, why, and to whom (Gustafson & Rice, 2020; Way, 2017).

Across the message attributes literature, risk communication research consistently demonstrated the paramount importance of evaluating risk messages (Downs, 2011; Fischhoff, 2019). Messages designed through intuition alone can easily cause unintended negative effects (Bryne & Hart, 2009; Hart, 2014; Hart & Nisbet, 2012). Salmon, Byrne, and Fernandez (2014) illustrate how messages can inadvertently draw an audience's attention to risky behaviors, create unnecessary worry and confusion, or cause individuals to discontinue feeling concerned when they are in danger, among other negative effects. For instance, a meta‐analysis of fear appeals found that the absence of efficacy information, which provides information about what people can do to reduce their exposure to risk, in high threat messages can result in people ignoring messages (Peters, Ruiter, & Kok, 2013). Untested messages, including those that are seemingly clear and uncontroversial, may even “boomerang,” where they generate “the opposite effects of what [was] intended” (Hart, 2014, p. 203). Fischhoff (2017) describes how a simple medicines leaflet pictogram of a red circle with a slash over a pregnant woman was interpreted by some to mean the product was a contraceptive, while others thought that pregnant women should avoid the product. Decisions over message attributes inevitably lead to tradeoffs as all messages will “engender some unintended effects on some audience members under some circumstances” (Balog‐Way & McComas, 2020; Salmon et al., 2014, p. 298). Specific message attributes such as reducing psychological distance, communicating uncertainty, or using humor are unlikely to achieve a messenger's risk communication goal by themselves (Schuldt et al., 2018). Evaluating messages therefore is critical to effective risk communication and minimizing unwanted effects (Downs, 2011; Fischhoff, 2019), or as Fischhoff (2012) commented when referring to medical treatments: “One should no more release untested communications than untested pharmaceuticals.” Evaluations do not need to be expensive or time‐consuming and extensive guidance now exists (Fischhoff, 2019; Fischhoff et al., 2011).

### 3.3 Audiences

Any risk communication message is filtered through the receiving audiences’ own selective lenses, with risk perceptions continuing to dominate the literature (see Siegrist & Árvai, 2020). Recent reviews particularly highlight the importance of context in influencing risk perceptions. In a systematic review and meta‐analysis of public perceptions of food and feed products derived from genetically modified organisms, Frewer et al. (2013) found that plant‐ versus animal‐related applications were perceived as more acceptable, risk perceptions and moral concerns were greater in Europe than North America and Asia, and both benefit and risk perceptions increased over time. By examining nationally representative survey data from 119 countries, Lee, Markowitz, Howe, Ko, and Leiserowitz (2015) found that understanding the anthropogenic causes of climate change was the strongest predictor of climate change risk perception, although other factors like basic education and climate literacy highlighted the need to develop nationally specific risk communication strategies. In reviewing the “natural” hazards risk perception literature, Wachinger, Renn, Begg, and Kuhlicke (2013) found that personal experience of hazards like floods, droughts, earthquakes, volcanic eruptions, wildfires, and landslides, and preexisting trust in authorities and experts had the greatest impact on public risk perceptions.

Across the literature, a variety of approaches for measuring different dimensions of risk perception were used. For Wilson, Zwickle, and Walpole (2019), this includes *general* (e.g., “How risky is X?”), *probability and consequences* (e.g., “How likely is it that X will occur?” and “How serious will the consequences be if X were to happen?”), and *affect only* (e.g., “How do you feel about X?”) approaches, as well as studies measuring perceived *probability only*, *benefit versus risk*, or some combination of approaches such as *affect* and *probability* or *general* and *affect*. Explorations of “risk‐as‐feelings,” including the role of affect and emotions, was arguably the most significant risk perception research development over the last decade (Skagerlund, Forsblad, Slovic, & Västfjäll, 2020; Slovic, 2010; Tompkins, Bjälkebring, & Peters, 2018; Västfjäll, Peters, & Slovic, 2014). A longitudinal study (Burns, Peters, & Slovic, 2012) on emotional reactions to the 2008 financial crisis, for instance, found that, through the process of hedonic adaptation, negative emotional reactions to the crisis subsided over time and were highly predictive of heightened risk perception. Risk perception research also particularly expanded on personal factors including gender (e.g., Yang, Khoo‐Lattimore, & Arcodia, 2017), race (e.g., Macias, 2016), age (e.g., Balog‐Way, Evensen, & Löfstedt, 2020; Greenberg, 2012), political orientation (e.g., Yang, Chu, Kahlor, 2019), and numeracy (e.g., Peters, 2012). For example, in examining the influence of various sociopolitical and demographic variables associated with climate change across 22 European countries and Israel, Poortinga, Whitmarsh, Steg, Böhm, and Fisher (2019) found that human values, political orientation, gender, age, and education were all important predictors of climate change risk perception, although findings from one country inconsistently generalized to other national contexts.

Efforts to better understand audiences’ risk perceptions led to a body of work examining “cultural cognitions.” This research “attempts to fuse” two preexisting theories—cultural theory^10^ and the psychometric paradigm^11^—to explain public disagreements over important risk issues ranging from nuclear power and guns to nanotechnology and vaccines (*see* Kahan, Jenkins‐Smith, & Braman, 2011, for an overview). In a survey study of U.S. adults (*N* = 1,500), Kahan et al. (2011) found a strong correlation between respondents’ cultural values and their perceptions of scientific consensus relating to climate change, nuclear waste, and handguns. Individuals holding hierarchical and individualistic “cultural outlooks” significantly disagreed with those holding egalitarian and communitarian outlooks (also see Kahan, Braman, Cohen, Gastil, & Slovic, 2010; Kahan, Braman, Slovic, Gastil, & Cohen, 2009; Kahan et al., 2012). Cultural cognition, however, has been critiqued with arguments often reflecting extant criticisms of cultural theory (cf. Boholm, 1996, 2018) and limitations of the psychometric paradigm (cf. Slovic, 2000). The main arguments are that the theory does not clearly define culture, conflating the concept with values, worldviews, and political ideology; underestimates the heterogeneity of “the public”; overgeneralizes or ignores findings from the wider risk communication literature such as the relation between trust and value similarity (e.g., Siegrist, 2019), and, perhaps most critically, suffers from circular reasoning fallacies, whereby its meaning derives primarily from its self‐referential nature (Persson, Sahlin, & Wallin, 2015; Price, Walker, & Boschetti, 2014; van der Linden, 2016; van Der Linden et al., 2017). Although debate continues (Johnson & Swedlow, 2019; Kahan & Carpenter, 2017), the hypothesis has certainly drawn much needed critical attention to the importance of culture and values in shaping audiences’ risk perceptions, while positively demonstrating the evolving nature of risk communication as a vibrant, diverse, and theoretically contested field (Moser, 2016).

A noteworthy outcome of cultural cognition discussions was an increased emphasis on the concept of motivated reasoning (Kahan, 2015a; 2015b; also see Chaiken, Giner‐Sorolla, & Chen, 1996). Rather than simply responding to the content of messages, it is clear that audiences’ cultural values and ideology may cause them to respond based on a salient identity. Motivated reasoning has been used to explain, for instance, why conservatives with relatively high science knowledge compared to those with low science knowledge are more likely to reject climate change (Kahan, 2015c). A closely connected research strand continued to explore the relationship between audiences’ behavior and support and messages aimed at changing various types of evaluative beliefs. Such beliefs represent core objectives of much communication (Dudo & Besley, 2016). This includes beliefs about risks and benefits (e.g., Mildenberger, Lubell, & Hummel, 2019), social norms (Rhodes, Shulman, & McLaran, 2020), and audience self‐efficacy (e.g., Bostrom, Hayes, & Crosman, 2019; Crosman, Bostrom, & Hayes, 2019; Dahlstrom, Dudo, & Brossard, 2012). Such variables are key elements of most health, environment, and risk communication behavior change theories (Montano & Kasprzyk, 2015). One particularly active debate has been about the degree to which messages focused on the presence of a scientific consensus can be used to gain support for climate change policy (Bolsen & Druckman, 2018; Dixon, Hmielowski, & Ma, 2019; Lewandowsky, Gignac, & Vaughan, 2013; van der Linden, Leiserowitz, & Maibach, 2019), and other issues such as genetically modified food (Dixon, 2016).

Other behavioral outcomes that factored frequently in risk communication research were audience's willingness to take protective actions and seek risk information. Among prominent theoretical frameworks, the Protective Action Decision Model (Lindell & Perry, 2012), originally posited to predict behavioral intentions in the face of “natural” disasters, such as hurricanes (Rickard et al., 2017b), wildfires (McCaffrey, Wilson, & Konar, 2018), and tornadoes (Miran, Ling, Gerard, & Rothfusz, 2019), was also used to examine infectious diseases (Johnson, 2019) and chemical spills (Heath, Lee, Palenchar, & Lemon, 2018). Efforts to understand information seeking behaviors frequently built on the Risk Information Seeking and Processing model (Yang, Aloe, & Feeley, 2014), which integrates messenger, message, and audience characteristics to predict information seeking behaviors. The last decade saw this model applied in many contexts, including air pollution in South Korea (Kim & Kim, 2019), crisis communication via social media (Sutton, Woods, & Vos, 2018), and the 2014 Ebola outbreak (Yang, 2019), suggesting the versatility of the framework. Furthermore, the model has recently been extended to consider not just different contexts but different behaviors such as public support for climate change mitigation policy (Yang et al., 2014).

The past decade also saw continued research on the processes and outcomes of public engagement (Besley, 2015; McComas et al., 2009; Webler & Tuler, 2018). Incorporating public participation, dialog, and deliberation, public engagement can be broadly conceptualized as a continuum ranging from low‐involvement “audience” activities like filling in a survey or signing a petition, to high‐involvement activities such as attending a series of meetings or sitting on an advisory board (Besley, 2015). At the high‐involvement end, a sizable literature continued to explore the benefits, limitations, and challenges of “upstream” engagement in the scientific and technological development process (Pidgeon, 2020). Studies capitalized on contemporary emerging technologies like climate engineering (e.g., Bellamy, Chilvers, Vaughan, & Lenton, 2013), future energy systems change (e.g., Demski, Spence, & Pidgeon, 2017), carbon capture and storage (CCS) (e.g., Thomas, Pidgeon, & Roberts, 2018), and unconventional oil and gas development (e.g., North, Stern, Webler, & Field, 2014). In the nanotechnology context, Pidgeon et al. (2017) found that, inter alia: (1) members of the general public are perfectly capable of debating complex issues when they are given sufficient resources, time, and support; (2) valuable benefit–risk perception knowledge can be obtained before social controversies occur; and (3) projected resistances ultimately depend on the way different types of nanotechnology are used.

Research also suggested that meaningful engagement remains fraught with challenges, impeding the promises of improving decision quality and achieving other normative, instrumental, and substantive imperatives. In the U.S. context, Webler and Tuler (2018) suggested that unfulfilled promises were driven by a scarcity of attitudes and aptitudes supportive of public participation, an antidemocratic atmosphere, decreasing oversight of private interests, and widespread distrust in institutions. Rothstein (2013) argues that some organizations have coped and adapted to public participation pressures rather than truly fostering meaningful engagement. Other challenges include: participants feeling they have little meaningful influence, or becoming disenchanted with the realities of “backstage” decision making; decisionmakers feeling pressurized, reducing their ability to discuss sensitive or complicated issues; organizers requiring substantial multistakeholder commitment and capacities; and, ultimately, engagement potentially exacerbating risk disputes (Besley, 2015; Löfstedt & Bouder, 2014; Pidgeon et al., 2017). Nevertheless, many argue that low and high involvement public engagement is here to stay (Löfstedt, Bouder, Wardman, & Chakraborty, 2011) and needed (Pidgeon, 2020), while providing beneficial impacts for participants (Jardine & Driedger, 2014; Karpowitz & Mendelberg, 2011). In providing constructive suggestions, Pidgeon (2020) discusses how the public engagement process should seek to (1) provide participants with balanced information and policy framings; (2) open and maintain deliberative spaces that enable different forms of engagement and reflection; (3) avoid naïve audience sampling strategies; and (4) use varied methods to elicit broader values. While practitioners are encouraged to engage with multidisciplinary research, there is equally a need to systematically and empirically test new ideas in order to advance current theories and fulfil the promises of meaningful public engagement (Besley, 2015).

## 4 CONCLUSION

The interdisciplinary field of risk communication continued to evolve over the last 10 years, building on decades of accumulated research and experience (cf. Fischhoff, 1995; Leiss, 1996; McComas, 2006). The boundaries of risk communication are not static but have been shaped and reshaped over time through a continuing series of historical events and processes. The field has been particularly influenced by evolving conceptualizations of “risk” in society and varying perspectives on how risk communication could or should be constituted in practice. Our takeaway is that efforts to settle on a single, generic version of what constitutes risk communication will be less productive than an open‐minded exploration of the multiple forms that comprise today's vibrant interdisciplinary field. Recognizing different forms does not mean that all arguments for engaging in multiway risk communication are acceptable, ethical, or effective in every circumstance. Some arguments are valid in some contexts, but certainly not in others. Herein lies a vital role for SRA and *Risk Analysis*. By providing open forums and new opportunities, the society and its flagship journal can proactively lead the way toward greater conceptual clarity about the continually evolving field, its boundaries, and future directions.

Reflecting on our review of risk communication messengers, message attributes, and audiences, four overarching findings are highlighted. First, over the last decade risk communication researchers continued to engage with important contemporary events and issues (e.g., COVID‐19), emerging technologies (e.g., nanotechnology), new communication channels (e.g., social media), and hypotheses (e.g., cultural cognition). However, themes identified in past reviews such as trust, framing, risk perception, and public engagement remain relevant to current challenges (cf. McComas, 2006). This demonstrates that researchers both continue to engage with contemporary developments while building on extant themes. Second, our review consistently suggests that effective risk communication requires a multifaceted approach. No simple or dominant formula for risk communication exists. The message attributes literature, for example, shows that reducing psychological distance, communicating uncertainty, or using humor alone is highly unlikely to achieve desired results; these are only a few among multiple attributes that influence message outcomes and messages rarely have direct effects on audiences but rather are typically contingent, indirect, and cumulative. Third, many concepts like trust, transparency, and uncertainty were found to be multidimensional and thus require more nuanced discussions than sometimes occur. Although many promulgated their virtues, risk communicators must recognize that different degrees exist, which each have varying positive, negative, and limited effects. Fourth, risk communication—recognized as an ongoing process and not a one‐time event—deserves and requires meaningful evaluation, long‐term commitment and, as Kasperson (2014) argues, “perseverance” in order to produce lasting, positive outcomes.

Our review also revealed that interdisciplinarity remains one of the field's greatest strengths, fueling significant new advances. Researchers have long drawn from a range of disciplines, and the last decade was no exception. Research on topics like transparency, the affective and emotional components of messages, and public engagement particularly advanced by applying theories from public administration (e.g., Hood & Heald, 2006), psychological dual processing models (e.g., Slovic et al., 2004), and democratic governance (e.g., Arnstein, 1969; Pateman, 1970), respectively. Emerging technologies such as fracking, nanotechnology, and autonomous vehicles stimulated research on public acceptance, while affording new contexts for exploring upstream public engagement (Pidgeon, 2020). Similarly, while climate change provided opportunities for exploring theories of psychological distance (e.g., construal level theory) (Trope & Liberman, 2010), contemporary crises such as Fukushima, Hurricane Sandy, and the COVID‐19 pandemic led to new research on warnings, the protective action decision model (Lindell & Perry, 2012), and uncertainty communication. Interdisciplinarity therefore continues to be a cornerstone of the field.

Even so, we see opportunities to nurture this interdisciplinarity further through cross‐fertilizing research. After all, interdisciplinarity was a fundamental reason for creating *Risk Analysis* in the first place, as explained by the first editor, Robert Cumming (Greenberg & Lowrie, 2011, p. 7):

peers were frustrated by the lack of access to ideas outside of their own discipline. Some, he told us, pointed to instances when their research would have benefited by crossing disciplines. Collectively, they urged Dr. Cumming to create a journal dedicated to interdisciplinary risk analysis.

Forty years into the journal's evolution, we concur that researchers should continue to draw from developments in other fields, including the full range of social sciences and humanities, to advance our understanding of risk communication.

Nurturing interdisciplinary research, however, will require addressing the tendency of the academic system to reward disciplinary over interdisciplinary scholarship. Early and midcareer researchers seem particularly pressurized to design studies, publish in journals, and seek grants that can progress their career in discipline‐focused departments. Most university faculty hiring and promotion committees, for example, require evidence of clear contributions that can progress the department's “home” discipline. *Risk Analysis*, the *Journal of Risk Research*, and various other scientific journals encourage interdisciplinary risk communication research. Most, however, cater to specific disciplines, with editors and reviewers often expecting certain studies and authors are cited, research questions asked, and methods used. Disciplinary territorialism also can result in scholars claiming that risk communication “belongs” to “their” discipline. There are, of course, departments, institutions, and other bodies that both encourage and reward interdisciplinary approaches. The Decision, Risk, and Management Sciences (DSRM) program at the U.S. National Science Foundation (NSF), for example, has multidisciplinary panels regularly evaluating promising interdisciplinary risk communication research. Yet, far more opportunities and a greater interdisciplinary appetite is needed across the academic system.

A second key challenge centers on the field's pragmatic function and especially the policy relevance of risk communication research. Despite substantial advances captured in this review, serious questions remain over the extent to which risk communication research has penetrated policy and practice. By interviewing officials at six government agencies in Sweden, Boholm (2019) found a substantial gap between the academic study of risk communication and government agency practice. Interviewees, for example, expressed “little or no effort to explore the actual concerns of members of the public” and “very little critical reflection” on the role of science in risk communication (Boholm, 2019, p. 1704). In his commentary, Kasperson (2014, p. 1234) argued that, although there have been successes, risk communication “seems little changed from practice decades ago” and the field “is replete with examples of missed opportunities, missteps, and outright failure”. Examples include the disposal of high‐level radioactive waste in the United States (Kasperson, 2014), the Hallandås railway tunnel crisis in Sweden (Boholm, 2009), and various international food scares relating to acrylamide, bisphenol A, and food colorings (Löfstedt, 2013a), to name a few. Perhaps more than any other event in recent memory, the COVID‐19 pandemic has underscored the sizable gap between risk communication research and practice in many, but certainly not all, countries and organizations. While some point to a “research‐policy gap” where evidence‐based recommendations are not reaching practitioners, others argue that risk communication research needs to keep pace with rapidly changing policy environments to become more policy relevant (Rickard, 2019; Wardman, 2008).

Rather than evaluating these claims, we conclude by offering two recommendations on how the academic risk communication community might better work with practitioners. First, we believe that our community needs to find ways to increase the number and scope of vehicles for connecting researchers and practitioners. Boundary organizations such as SRA, the American Association for the Advancement of Science (AAAS), the National Academy of Sciences, Engineering, and Medicine (NASEM), and the U.K. Royal Society are dedicated to “connecting worlds that need one another, but do not normally interact” (Fischhoff, 2020, p. 139; O'Mahony & Bechky, 2008). During the COVID‐19 pandemic, for example, NASEM published various rapid expert consultations, including: *Encouraging the Adoption of Protective Behaviors to Mitigate the Spread of COVID‐19* (Brossard, Wood, Cialdini, & Groves, 2020); and the *Effectiveness of Fabric Masks for the COVID‐19 Pandemic* (Besser & Fischhoff, 2020). The risk communication community needs to capitalize on opportunities afforded by these boundary organizations. Such national‐level organizations, however, are still too distant from many practitioners. Therefore, new and more targeted vehicles are needed. Examples of bodies established over the last decade in Europe and the United States include the Informal European Parliamentary Working Group on Risk (see Löfstedt, 2013b), the FDA's Risk Communication Advisory Committee (Fischhoff, 2017, pp. 121–123), and the Ditchley Group Forums on benefit–risk communication (Löfstedt & Bouder, 2012), all of which hold targeted meetings with a range of academics, practitioners, and other stakeholders working at the coal‐face of risk communication. Although working with boundary organizations can be time‐consuming and challenging, they can be powerful vehicles for productively connecting researchers and practitioners to improve policymaking (see O'Mahony & Bechky, 2008).

Second, understanding policy relevance should become a core area of risk communication research. Inasmuch as evidence is needed on the practice of risk communication, so too is evidence needed on how risk communication research and practice can build on each other. In a systematic review, Oliver, Innvar, Lorenc, Woodman, and Thomas (2014) found that timely access to good quality evidence, collaboration with policymakers, and relationship‐ and skills building were the most important factors influencing the use of evidence. In his book, *The Politics of Evidence‐Based Policy Making*, Paul Cairney (2016) explains how simplistic policymaking assumptions can create confusion about, for instance, who “the policymakers” are, where “they” obtain influential evidence, and how decisions are made. For example, many academics assume that a small number of policymakers control the policy process, when power is really “shared across many government departments, levels of government, and with a range of quasi‐governmental and non‐governmental actors” (Cairney, 2016, p. 15). What is needed is a research program dedicated to understanding local, national, and international risk communication contexts (cf. Oliver & Cairney, 2019). Evidence‐informed policy engagement can help guide the way toward improving the field's policy relevance and communicating risk communication research more effectively.

## Footnotes

1 Articles were identified by reading the titles, keywords, and abstracts of every article published in *Risk Analysis* between January 2010 (volume 30, issue 1) and December 2019 (volume 39, issue 12). Others may arrive at different estimates, partly depending on varying conceptualizations of what counts as “risk communication” research. Nevertheless, the authors believe this simple method provides a helpful general estimate of the quantity of risk communication articles published in the journal over the decade.

2 This underscores the importance of boundary organizations like SRA, which bring together academics and practitioners to discuss these linkages.

3
*See* earlier developments in the field including the first 30 years since SRA's establishment (Fischhoff, 1995; Leiss, 1996; McComas, 2006; Plough & Krimsky, 1987); concepts like trust (Earle, 2010; Siegrist, 2019), culture (Johnson & Swedlow, 2019), and uncertainty (Löfstedt & Bouder, 2017); methods such as mental models (Boase, White, Gaze, & Redshaw, 2017; Bruine de Bruin & Bostrom, 2013), and risk perception measures (Wilson, Zwickle, & Walpole, 2019); and the underlying rationales and theoretical orientation of the field (Wardman, 2008; Rickard, 2019). Other reviews focused on specific risks and risks issues in a wide range of policy domains and contexts including health (McComas, 2006), food safety (Frewer et al., 2016), pharmaceuticals (Way, Blazsin, Löfstedt, & Bouder, 2017), climate change (Moser, 2016), and “natural’ hazards/disasters” (Thompson, Garfin, & Silver, 2017; Wachinger et al., 2013).

4 Many use the term “two‐way” risk communication. However, “multi‐way” signifies how many individuals, groups, and organizations have a stake and are involved in risk communication processes (McComas, 2006).

5 For Rickard (2019, p. 1), risk communication has two fundamental functions: “(1) a pragmatic function, in which senders direct messages at audiences (and vice versa), with various intended (and sometimes unintended) effects; and (2) a constitutive function, in which messages re(create) what we mean by ‘risk’ in a given social context, including how we can, and/or should relate to it.”

6 Some authors may move items from one subsection to another; change the labels to, for instance, sources, content, and targets; or add new subsections/delineations altogether. For example, affect and emotion research could be categorized under the subcategory of audiences (rather than message attributes). In this example, however, the authors chose to focus on how research on affect and emotions has been used to improve the attributes of risk communication messages.

7 Although regulatory agencies and government administrations are the foci, research on information channels like social media are included (see Dunwoody & Griffin, 2014, for a discussion). In addition, Rickard (2019) notes that while some messengers are trained in risk communication or a related field, others inherit the task “by happenstance or necessity.”

8 In this article, trust refers to social trust: “the willingness to rely on those who have the responsibility for making decisions and taking actions related to the management of technology, the environment, medicine, or other realms of public health and safety” (Siegrist, Cvetkovich, & Roth, 2000, p. 354). The “social” adjective emphasizes the class of people being trusted, that is, messengers with formal responsibilities for risk communication like governments and regulatory agencies.

9 Many other goals and reasons for transparency have been examined, however, for many messengers the ultimate goal is to build public trust (see Balog‐Way, Evensen, Löfstedt, & Bouder, 2019).

10 For a discussion of cultural theory, see Douglas and Wildavsky (1982), Wildavsky and Dake (1990), Dake (1991), Rayner (1992), Boholm (1996), and Johnson and Swedlow (2019).

11 For a discussion of the psychometric paradigm see Slovic (2000).
